# Hemi-central retinal artery occlusion in young adults

**DOI:** 10.4103/0301-4738.67069

**Published:** 2010

**Authors:** Pukhraj Rishi, Ekta Rishi, Tarun Sharma, Sheshadri Mahajan

**Affiliations:** Shri Bhagwan Mahavir Vitreoretinal Services, Sankara Nethralaya, 18, College Road, Chennai-600 006, Tamil Nadu, India

**Keywords:** Central retinal artery occlusion, Eisenmenger’s syndrome, embolus, hemi-central artery occlusion, Leiden mutation, malignant hypertension, polycythemia, retinal artery occlusion, thrombosis

## Abstract

Amongst the clinical presentations of retinal artery occlusion, hemi-central retinal artery occlusion (Hemi-CRAO) is rarely described. This case series of four adults aged between 22 and 36 years attempts to describe the clinical profile, etiology and management of Hemi-CRAO. Case 1 had an artificial mitral valve implant. Polycythemia and malignant hypertension were noted in Case 2. The third patient had Leiden mutation while the fourth patient had Eisenmenger’s syndrome. Clinical examination and fundus fluorescein angiography revealed a bifurcated central retinal artery at emergence from the optic nerve head, in all cases. Color Doppler examination of the central retinal artery confirmed branching of the artery behind the lamina cribrosa. It is hypothesized that bifurcation of central retinal artery behind the lamina cribrosa may predispose these hemi-trunks to develop an acute occlusion if associated with underlying risk factors. The prognosis depends upon arterial recanalisation and etiology of the thromboembolic event.

Acute retinal arterial obstruction presents as central retinal artery (CRA) obstruction in 57% cases, branch retinal obstruction in 38% and cilioretinal artery obstruction in 5%.[1] It may be related to known preexisting systemic disease or may be an initial manifestation of previously undiagnosed systemic abnormality. In young adults with retinal artery occlusion, associated etiological factors are more often obscure and diverse.[2] Hemi-central retinal artery occlusion (Hemi-CRAO) is an extremely uncommon clinical entity that has hardly been described in the literature. Hereby, we describe systemic and ophthalmologic characteristics of four patients ranging between 22 and 36 years of age, who presented with hemi-central retinal artery occlusion.

## Case Reports

### Case 1

**Figure 1:**
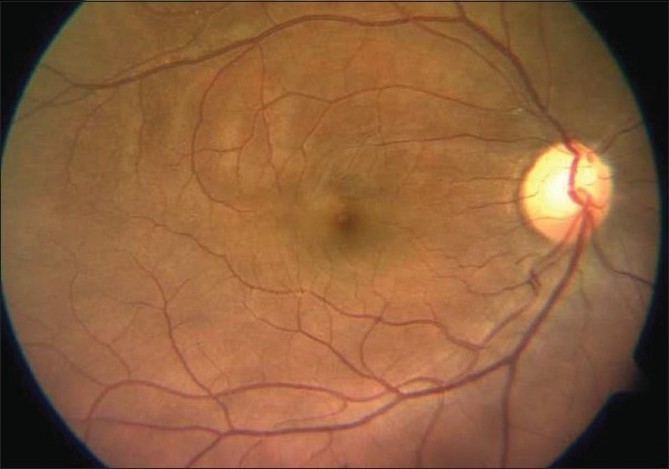
Case 1. Color fundus photograph of the right eye shows attenuation of the superior and supero-temporal retinal arterioles. Retinal pigment epithelial alterations are noted in the macula

**Figure 2:**
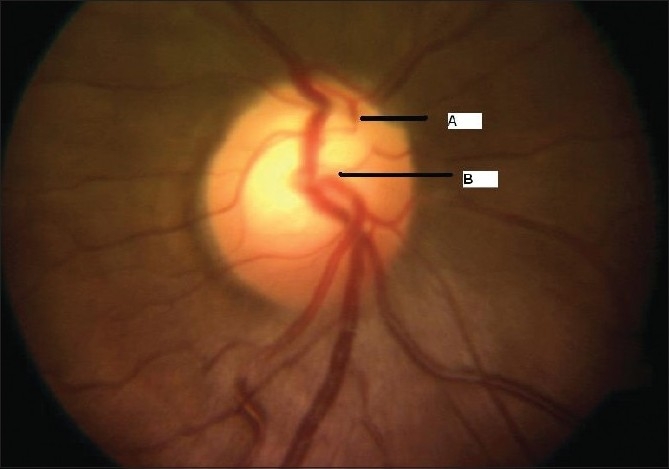
Case 1. Color fundus photograph of the optic nerve head shows the superior (A) and inferior (B) hemi-trunks of central retinal artery emerging separately

**Figure 3:**
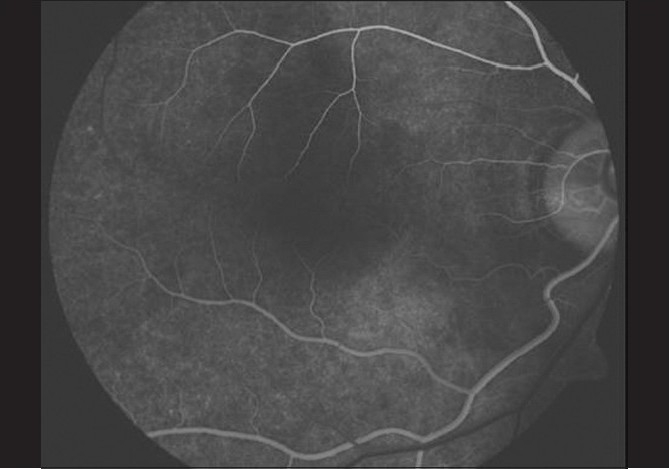
Case 1. Early phase FFA shows superior hemi-trunk of central retinal artery with a reduced arteriolar caliber and filling-in earlier as compared to the inferior hemi-trunk

**Figure 4:**
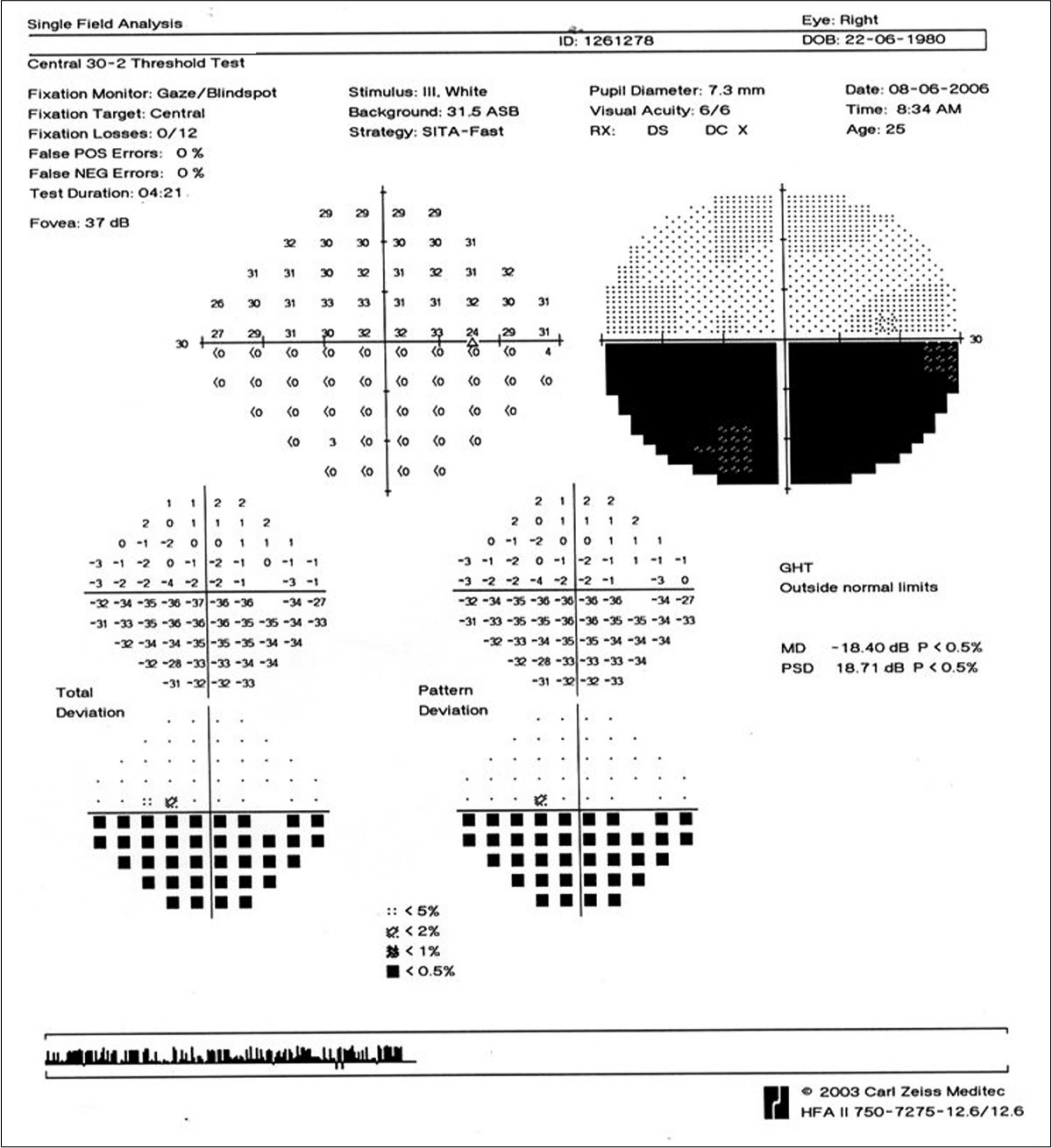
Case 1. A (30-2) Humphrey’s visual field analysis of the right eye shows an inferior altitudinal defect corresponding to the territory of vascular occlusion

**Figure 5:**
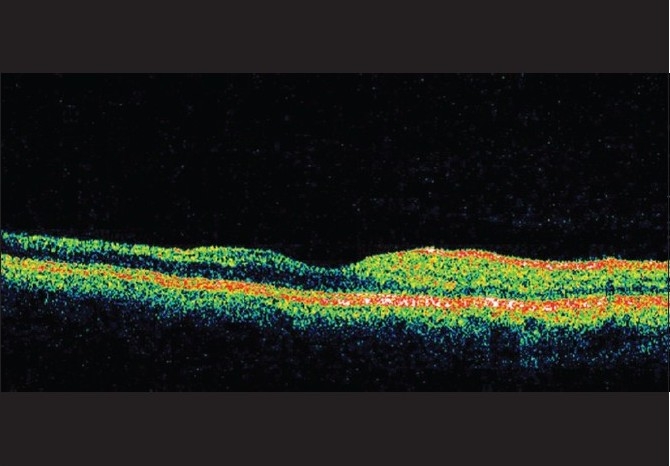
Case 1. Right eye OCT revealed retinal thinning of the superior half of the macula as compared to inferior half. Also noteworthy is the preferential loss of inner retinal layers. Both these findings correspond to the level and territory of retinal vascular occlusion

**Figure 6:**
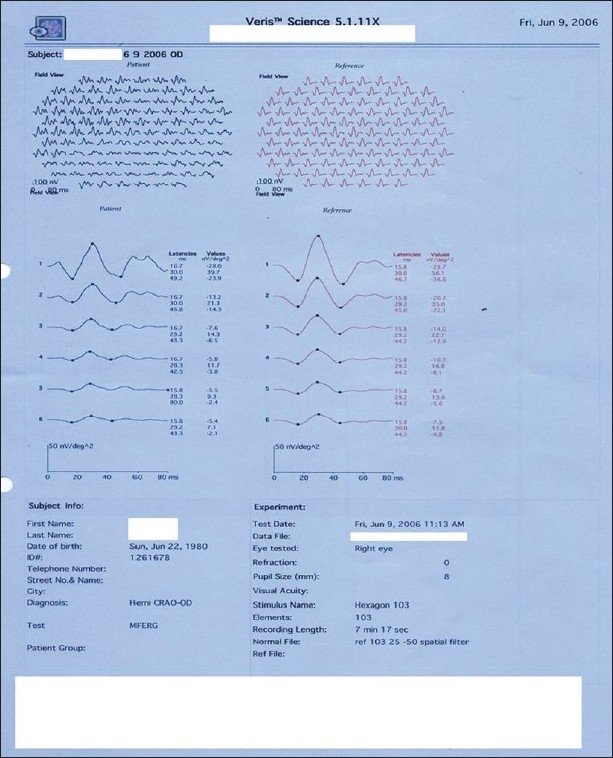
Case 1. Multifocal ERG of right eye showed normal implicit times and reduced amplitudes. This corresponds to the underlying pathology of retinal vascular occlusion

**Figure 7:**
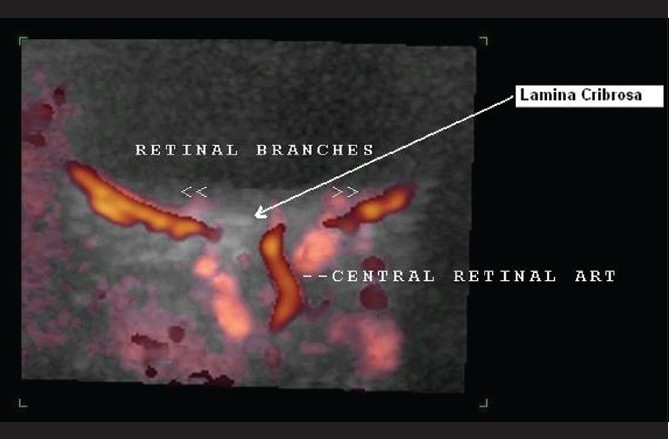
Case 1. Color Doppler study of the right eye shows branching of the central retinal artery into hemi-trunks just behind the lamina cribrosa (long arrow)

A 26-year-old gentleman presented with sudden, partial visual field loss in right eye. He had a prosthetic mitral valve implanted, 14 years back. Vision was 20/20 in both eyes. Anterior segment was essentially normal. Right fundus [Fig. 1] showed two central retinal artery hemi-trunks instead of main central artery [Fig. 2]. Supero-temporal arteriolar attenuation was noted; upper hemi-trunk was shorter. Retinal pigment epithelial alteration was noted; foveal reflex was dull. Left eye was normal. Findings of fundus fluorescein angiography (FFA) [Fig. 3], visual field examination [Fig. 4], optical coherence tomogram (OCT) [Fig. 5], multifocal electroretinogram (mfERG) [Fig. 6] and color Doppler study of right eye [Fig. 7] are summarized in Table 1. A summary of systemic investigations and treatment is included in Table 2. A cardiology consult was sought; patient was advised to continue current treatment [Table 2] with regular follow-up.

**Table 1:** Summary findings of Ocular Investigations

FFA features	Best Corrected Visual Acuity (BCVA) at presentation	ERG / multifocal ERG	Visual field	Color Doppler	Final VA
Earlier filling of superior herni-trunk with overall delayed arteriolar filling. Normal foveal perfusion	20/20	Normal implicit time and reduced amplitudes	Inferior hemispheric defect	Proximal post- lamina cribrosal branching of central retinal artery	20/20
Earlier filling of superior herni-trunk with overall delayed arteriolar filling- Wedge-shaped early hyperfluorescence with late staining and leakage in infero-temporal quadrant at the choroidal level.	No perception of light	Negative- negative scotopic waveform, loss of oscillatory potentials	Three quadrant defect	Proximal post- lamina cribrosal branching of central retinal artery	20/20
Blocked choroidal flush was seen superonasally.					
Delayed A-V* transit in superior herni-trunk; diffuse leak in involved area	20/60	Reduced amplitude in central and inferior field	Not done	Proximal post- lamina cribrosal branching of central retinal artery	20/60
Earlier filling of superior herni-trunk	20/20	Reduced b- wave response	Inferior hemispheric defect	Proximal post- lamina cribrosal branching of central retinal artery	20/20

A-V^*^: Arterio-venous

**Table 2:** Summary findings of Systemic Investigations and Management

Diagnosis (New/Known)	Investigations	Treatment (Initiated/Ongoing)
Prosthetic mitral valve (Known): 14 years back. Had episodes of momentary visual loss in right eye affecting the inferior field since last 2 years, which he ignored.	Hb*: 15.1 gm%, FBS†: 86 mg% S. lipid profile:normal. PT‡: 26 seconds. INR§: 5.04. Color Doppler: Bilateral major carotid vessels were normal. 2D color Doppler echocardiography: normal including normal movements of prosthetic mitral valve.	Oral Acetylsalicylate 75 mg/ day, Oral Acenocoumarol 2 mg/ day, Inj Benzathine Penicillin 12 lac Units I.M. once monthly, and Oral Pentoxyphylline 400 mgyday (Ongoing)
Polycythemia vera and Malignant HT (New) Sudden visual loss in left eye since four hours associated with headache and ocular pain.	Carotid pulse was well-felt, bilaterally. BP was 190/120 mmHg. Heart sounds were normal; no neurological deficit was evident. ECG║: normal sinus rhythm and LVH††. 2D Echocardiography: mild concentric LVH††. Hb 23.6 gm%, Total Erythrocyte count 7.3 million/mm^3^, ESR** 21 mm/ 1h (Westergren), Reticulocytes 1.5%. MCV 97 µm^3^, MCH 32 pg, MCHC 33%, normal peripheral blood smear findings; all suggestive of polycythemia. Plasma homocysteine: 6.6pnVL, Serum erythropoeitin 3.6 mU /ml. Hb electrophoresis: no abnormal band. Arterial oxygen saturation: 99%. RPR‡‡ test: Non-reactive. Anti-HIV I and II antibodies were not detected.	At presentation: Ocular massage was started immediately. 500 mg oral acetazolamide was administered and topical timolol 0.5% was applied. Definitive treatment: Oral antihypertensives and serial phlebotomies (Initiated).
Factor V Leiden mutation (New), HT, mild MR and chorea (Known) Spontaneous abortion 4 months back.	B.*P*. = 110/80 mmHg. Color Doppler study of carotid and vertebral arteries on both sides was normal. Mild mitral valve regurgitation was noted on echocardiogram. Hemoglobin 12.4gm%, 1h ESR 23mm (Westergren); Complete blood count, coagulation profile and serum lipid profile were normal. FBS†: 82mg/dl, TSH 4.4 µU/ml, Free T4 1.0 ng/dl. Free T3 0.34ng/ dl, FIA factor < 10 lU/ml, Positive antinuclear antibodies in primary dilution (1:40) along with speckled ANA pattern and 1+ immunofluorescence intensity by Serum antinuclear AB-IFA, HEP2, negative Serum ANCA, normal serum double-stranded DNA antibodies and serum ACE levels. A high protein C activity of 146% (reference level 70-130%) was found. Real time PCR detected Factor V Leiden mutation. Ornithine aminotransferase level was normal. Lupus anticoagulant test and anti-cardiolipin IgG and IgM antibody test were negative. Renal function tests: normal. Monteux test: negative.	Oral losartan, hydrochlorothiazide, sodium valproate and haloperidol (Ongoing)
Eisenmenger syndrome (Known) He had a history of mild chest pain, breathIessness, palpitation and giddiness. He had no history of essential hypertension, diabetes mellitus, trauma, drug abuse, cough, hemoptysis, syncope or swelling of feet.	Systemic examination revealed mild cyanosis and clubbing. Pulse rate was 86/min, blood pressure 106/70 mmHg. Hb*: 16.8gm%. ESR 3mm/1 h (Westergren). All coagulation indices normal. Plasma homocysteine: 14.0 µM by ELISA. Renal and liver function tests: normal. 2-D Echocardiography detected a large (18 mm), subaortic ventricular septal defect with bi- directional flow, severe pulmonary arterial hypertension and dilated right atrium, right ventrical and pulmonary artery; mild prolapse of tricuspid leaflet with normal interatrial septum. Normal left and right ventricular function was noted. Normal systemic and pulmonary venous drainage along with normal aorta and pericardium were noted.	Oral acetylsalicylic acid 50 mg daily. (Initiated) Heart-lung transplantation advised

Hb^*^: Hemoglobin

FBS^†^: Fasting blood sugar

PT^‡^: Prothrombin time

INR^§^: International normalized ratio

ECG^║^: Electrocardiogram

LVH^††^: Left ventricular hypertrophy

ESR^**^: Erythrocyte sedimentation rate

RPR^‡‡^: Rapid plasma region

### Case 2

**Figure 8:**
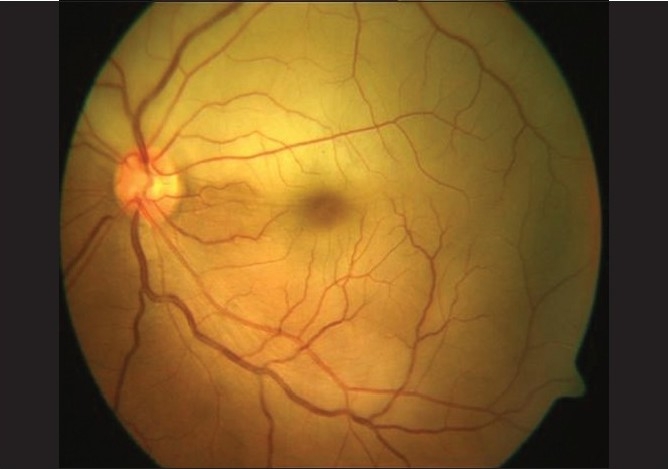
Case 2. Left eye fundus photograph reveals well-demarcated retinal edema of the superior quadrant and an accentuated foveal reflex

**Figure 9:**
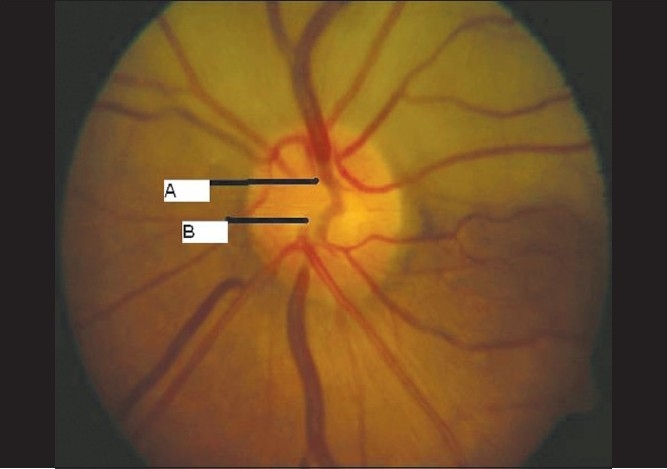
Case 2. Color fundus photograph of the optic nerve head shows the superior (A) and inferior (B) hemi-trunks of central retinal artery emerging separately

**Figure 10:**
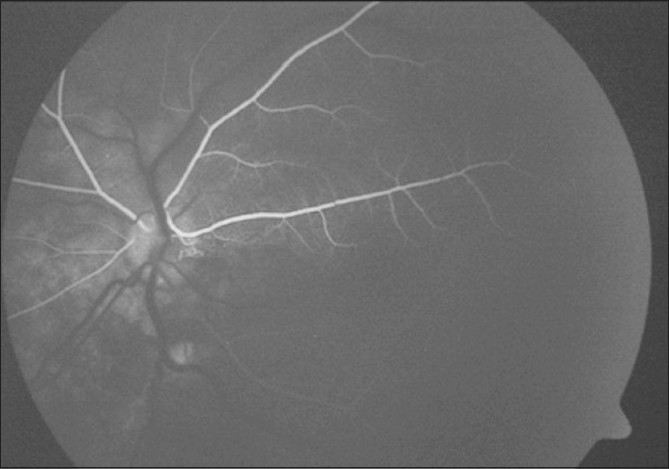
Case 2: Left eye FFA shows early filling of superior hemi-trunk

**Figure 11:**
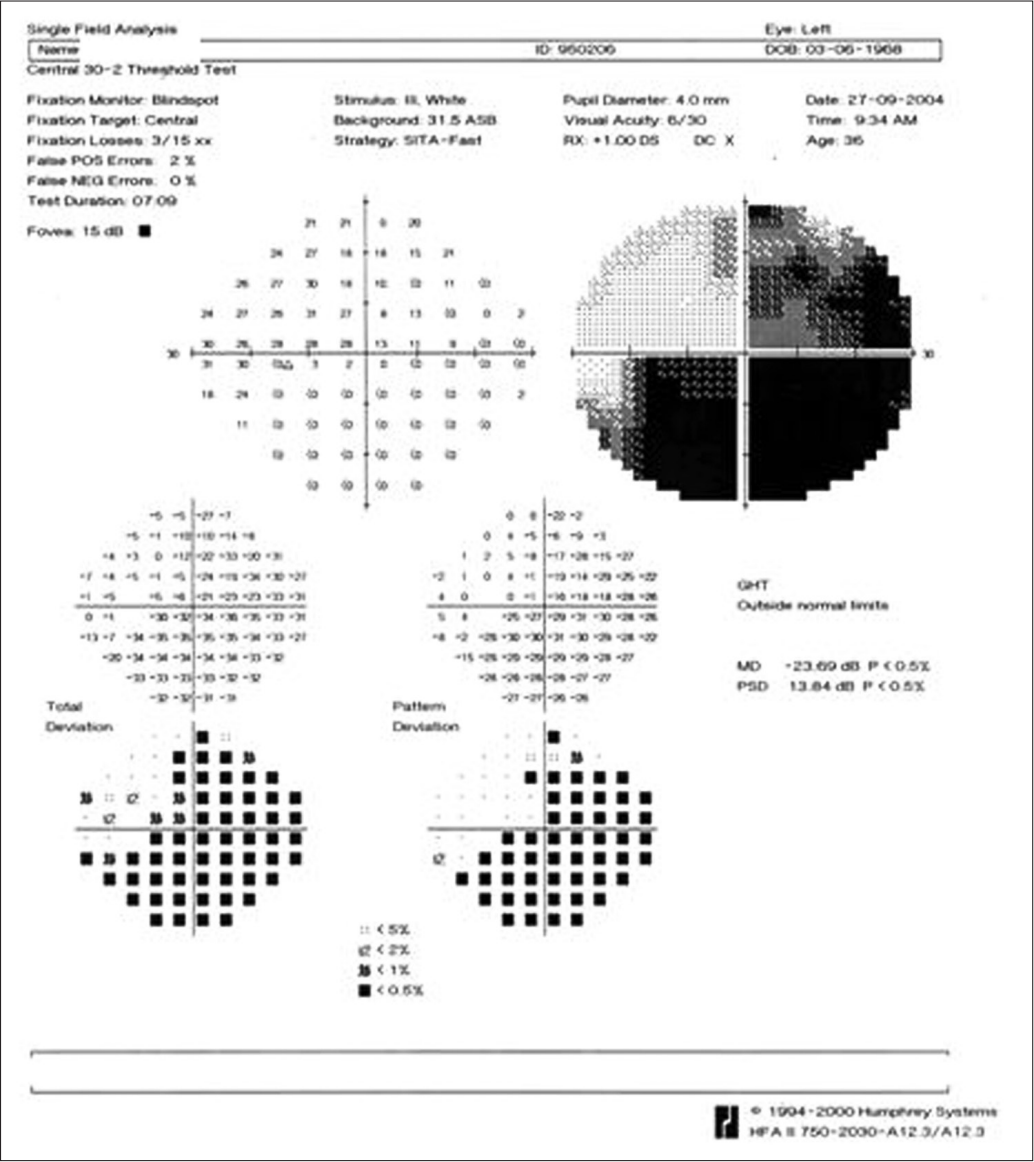
Case 2: Left eye visual field analysis shows an extensive defect corresponding to the territory of vascular occlusion

**Figure 12:**
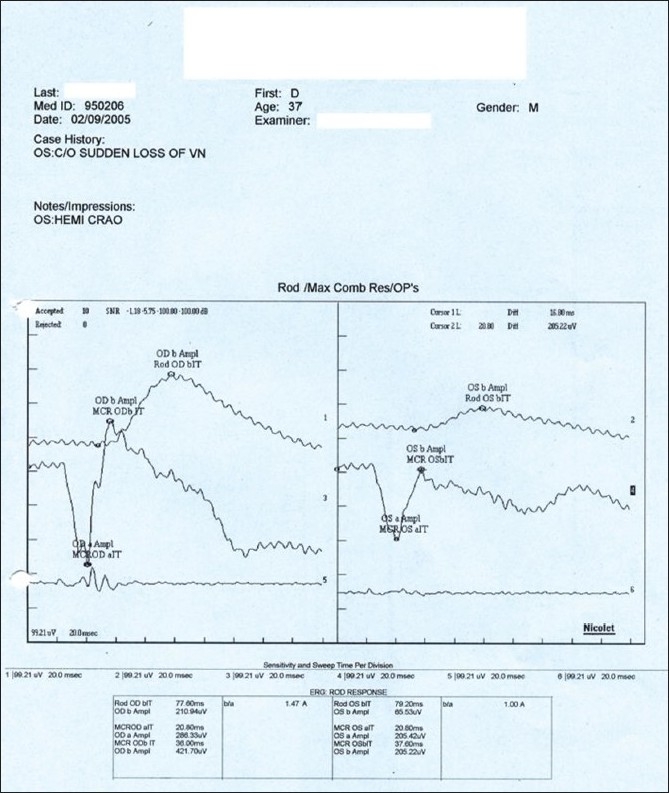
Case 2: Negative-negative waveform for scotopic response and a loss of oscillatory potentials were recorded in the ERG for the left eye. This corresponds to inner retinal ischemia consequent to retinal vascular occlusion

A 36-year-old gentleman reported with sudden visual loss in left eye. He had a similar problem two months back with spontaneous improvement, for which he did not seek any treatment. Vision was 20/20 in right eye and no perception of light in left. Anterior segment examination was normal. Right eye was normal. Left fundus revealed patchy areas of retinal edema over posterior pole and bifurcated central retinal artery [Figs. 8 and 9]. Generalized arteriolar attenuation, box-carring of vessels and cherry red spot at fovea were seen. Treatment was initiated immediately [Table 2]. However, fundus appearance remained same. He was referred to the physician. Next day, the patient reported perception of light in that eye. A platelet-fibrin embolus could be made out in infero-temporal arcade. Findings of FFA [Fig. 10 a and b], visual field examination [Fig. 11], ERG [Fig. 12] and color Doppler study of the right eye are summarized in Table 1. A summary of systemic investigations and treatment is included in Table 2. Five days later, vision recovered to 20/125. On the tenth day, it improved to 20/40 and after six months it was 20/20. The patient also had regular follow-ups with the hematologist and cardiologist.

### Case 3

**Figure 13:**
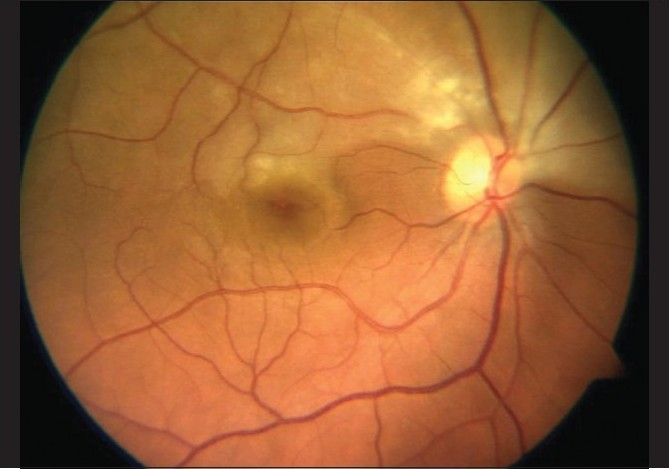
Case 3: Color fundus photograph of the right eye shows gross retinal edema involving the superior half of the fundus, albeit sparing the fovea

**Figure 14:**
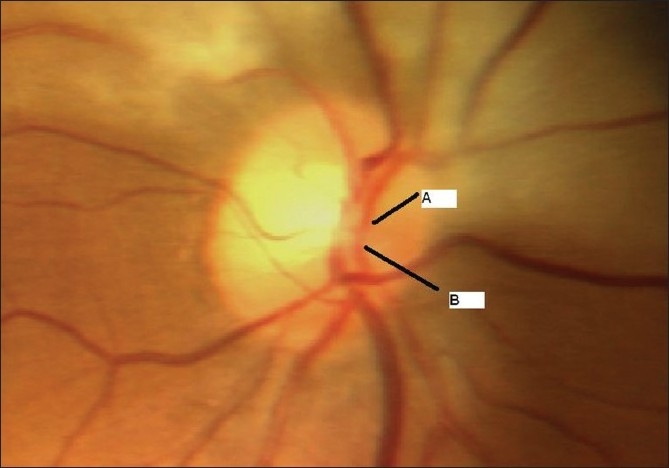
Case 3. Color fundus photograph of the optic nerve head shows the superior (A) and inferior (B) hemi-trunks of central retinal artery

**Figure 15:**
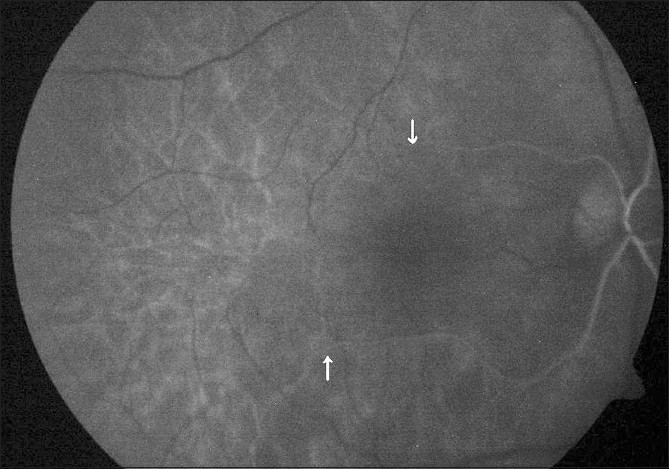
Case 3. Right eye FFA showed increased transit time along the superior retinal sector

**Figure 16:**
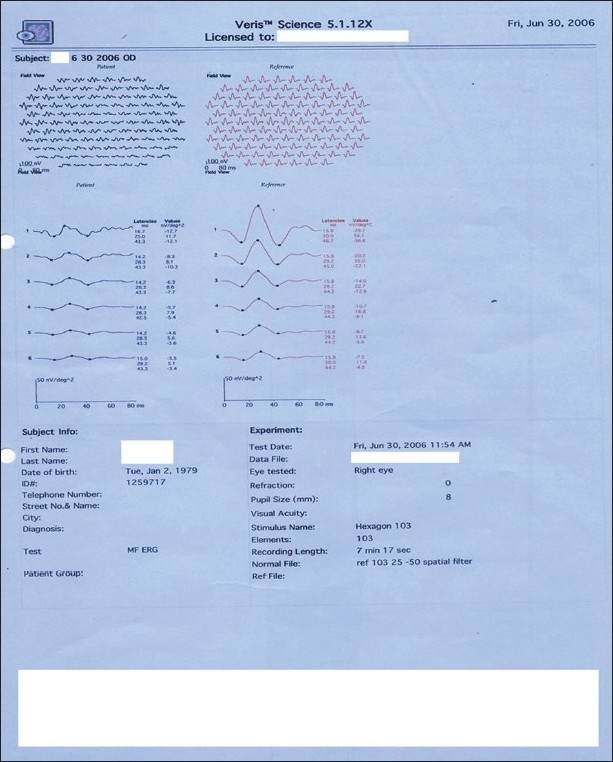
Case 3. Right eye multifocal ERG shows grossly reduced responses

**Figure 17:**
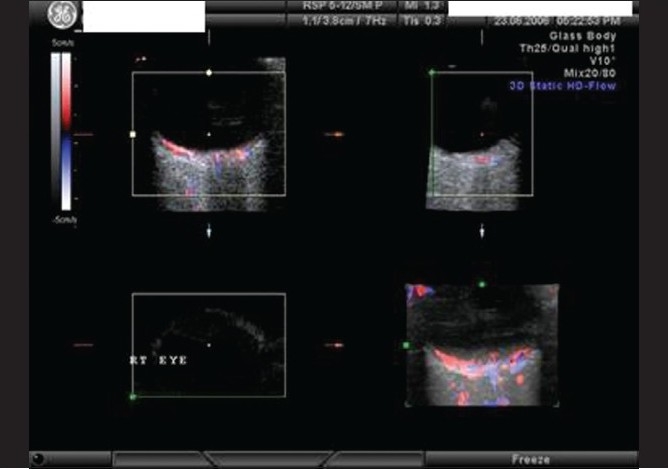
Case 3. Color Doppler of the right ophthalmic artery shows branching of the central retinal artery behind the lamina cribrosa

A 28-year-old lady reported with sudden, painless visual loss in right eye since five days. Diagnosed to have systemic hypertension, rheumatic mitral valve regurgitation and chorea, she was under treatment for the same [Table 2]. Vision was 20/60 in the right eye and 20/20 in the left. Anterior segment examination was unremarkable. Right fundus revealed retinal edema involving superior half, sparing fovea with no embolus [Fig. 13 and 14]. Left eye was normal. Findings of FFA [Fig. 15], multifocal ERG [Fig. 16] and color Doppler study of the right eye [Fig. 17] are summarized in Table 1. A summary of systemic investigations and treatment is included in Table 2. The patient was detected to have Factor V Leiden mutation (Real time Polymerase Chain Reaction, RT PCR) and was also advised a regular follow-up with cardiologist and hematologist.

### Case 4

**Figure 18:**
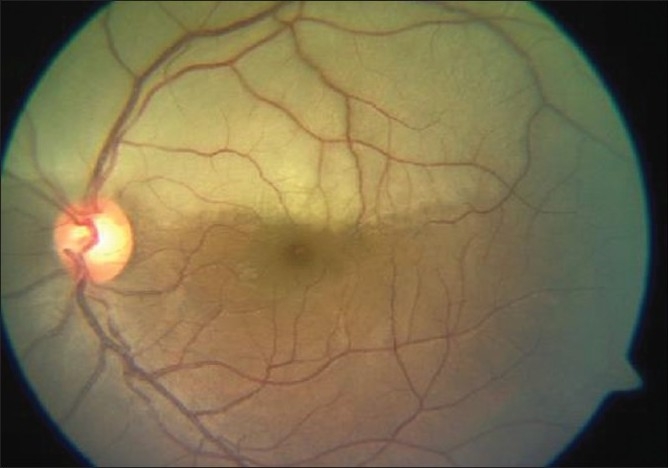
Case 4. Color fundus photograph of the left eye shows extensive retinal edema in the superior half, sparing the fovea

**Figure 19:**
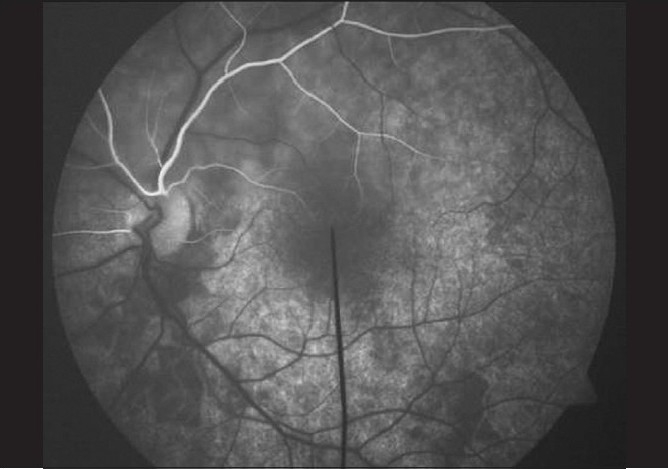
Case 4. FFA of the left eye shows early filling of the reperfused superior hemi-trunk

**Figure 20:**
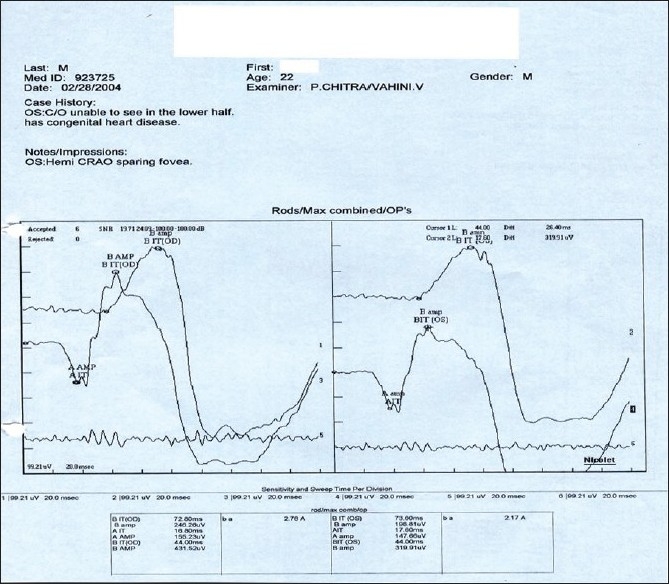
Case 4. Left eye ERG reveals a normal ‘a’ wave response and a reduced ‘b’ wave. This corresponds to inner retinal ischemia consequent to retinal vascular occlusion

**Figure 21:**
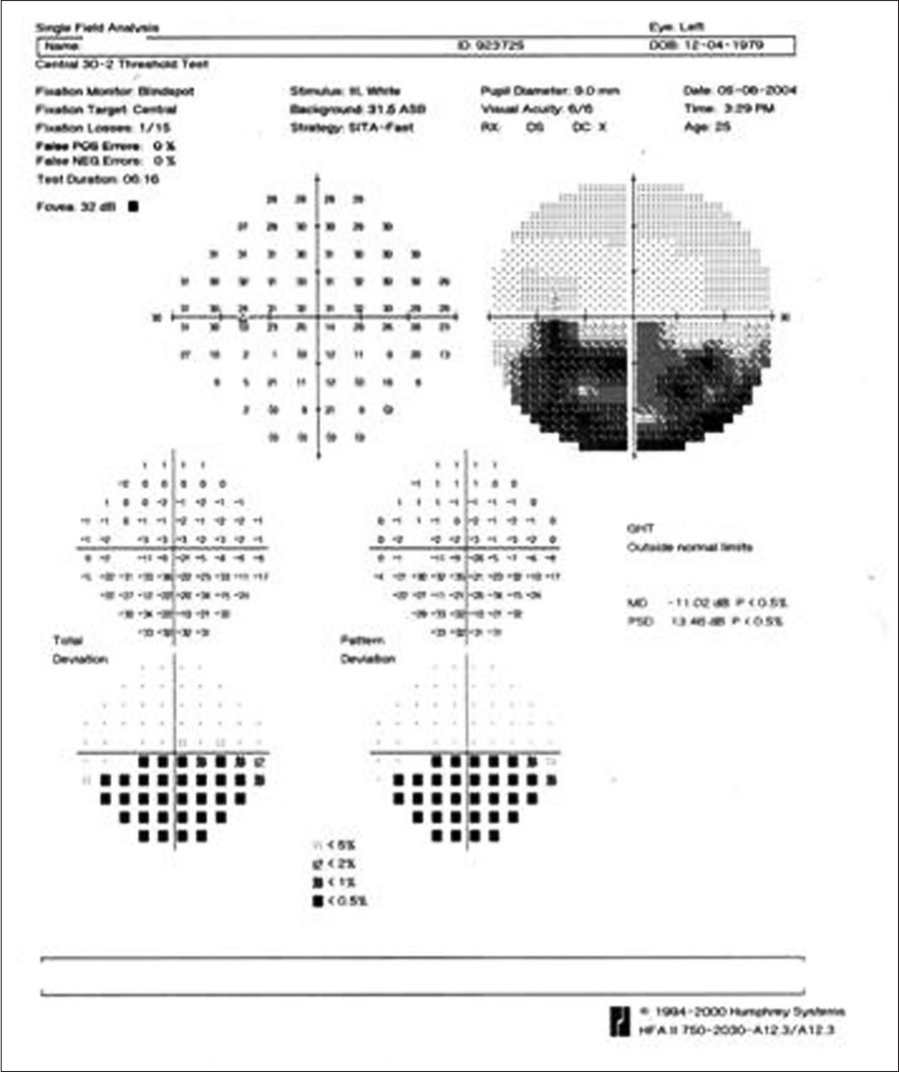
Case 4. Left eye visual field analysis reveals an inferior altitudinal defect corresponding to the affected retina

**Figure 22:**
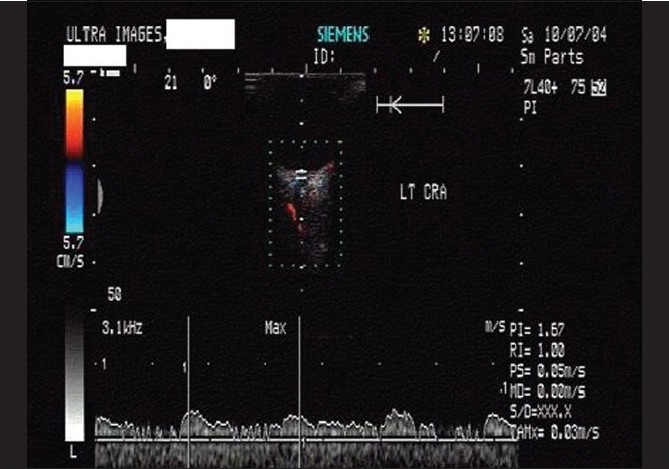
Case 4. Color duplex imaging of orbit detected a temporal branch emerging from central retinal artery before lamina cribrosa

A 22-year-old young man, a known case of congenital heart disease, reported with decreased vision in left eye since two days. Vision was 20/20 in both eyes. Anterior segment examination was essentially normal. Right fundus was normal. Left fundus revealed retinal edema involving superior half, sparing fovea with no embolus [Fig. 18]. Findings of FFA [Fig. 19], ERG [Fig. 20], visual field examination [Fig. 21] and color Doppler study of the right eye [Fig. 22] are summarized in Table 1. A summary of systemic investigations and treatment is included in Table 2. Diagnosed to have Eisenmenger syndrome, the probable cause of vascular occlusion was paradoxical embolism through ventricular septal defect. At six weeks review, retinal edema had reduced with no evidence of anterior segment neovascularisation.

## Discussion

CRA commonly originates as a separate stem from the first part of the ophthalmic artery and usually divides into two branches at the disc, each of which further bifurcates into temporal and nasal divisions. Anatomical variations of branching patterns are known.[3] Reports describe a case with two CRAs running independently up to the optic disc and joining at the optic disc to form a loop, from the summit of which arose terminal branches that followed the usual course.[4] However, this anatomical arrangement was not seen in any of our cases. Singh and Dass also describe a branch of CRA originating from behind the lamina cribrosa that supplied the supero-medial part of the retina but this was more of a ‘branching’ than a ‘bifurcation’.[5]

On entering the eye, CRA loses the elastic lamina and has a prominent muscularis as it bifurcates at the optic disc. These histological changes distinguish retinal arteries from muscular arteries of the same size in other tissues. Additionally, the unusually developed muscularis may allow greater constriction of the vessels in response to chemical and pressure changes.[6] These histological changes coupled with the pre-lamina cribrosa branching of CRA may make these hemi-trunks more ‘vulnerable’ to vascular occlusion, especially in subjects with systemic co-morbid conditions. Hence, hemi-CRAO may be a manifestation of a preexisting systemic condition or a harbinger of a hitherto undiagnosed systemic condition.

Thromboembolic events are the main culprits in the pathogenesis of retinal artery occlusions which can manifest as ‘Hemi-CRAO’ in a proximally bifurcated CRA.[7,8] A sole report has described hemi-CRAO occurring in association with sexual activity and sildenafil citrate; a coincidental finding as mentioned by the author.[9] In our series, Case 1 had an artificial mitral valve and was on anticoagulation therapy. Case 2 had polycythemia. Both are well documented etiological factors,[10–12] which lead to the devastating vascular episode. Genetic mutation in Factor V renders it resistant to anticoagulant effect of endogenous anticoagulant protein C. The most common of these mutations is called the Leiden mutation.[13] Similar etiological factor was reported in a 25-year-old woman with multiple bilateral retinal arteriolar occlusion[14] and in a 33-year-old lady with branch retinal vein occlusion.[15] Case 3 had Leiden mutation. There have been isolated case reports of ocular ischemic features in association with Eisenmenger’s syndrome.[16,17] However, its manifestation as hemi-CRAO (Case 4 in our series) is reported as first of its kind. All patients in this unique case series were young, had angiographic evidence of hemi-CRAO owing to proximal, pre-lamina cribrosal branching pattern of CRA (confirmed by color Doppler) and also had remarkable visual recovery of 20/60 or better.

To conclude, unusual pre-lamina cribrosa (extraocular) branching pattern of central retinal artery coupled with the unique histological features of the retinal arterioles (hemi-trunks) may predispose to the development of ‘hemi-central retinal artery occlusion’ in young adults with underlying systemic conditions. However, further histopathological studies are required to understand this clinical entity better.
